# Comparative genome sequencing reveals chemotype-specific gene clusters in the toxigenic black mold *Stachybotrys*

**DOI:** 10.1186/1471-2164-15-590

**Published:** 2014-07-12

**Authors:** Jeremy Semeiks, Dominika Borek, Zbyszek Otwinowski, Nick V Grishin

**Affiliations:** Molecular Biophysics Program and Medical Scientist Training Program, University of Texas Southwestern Medical Center, Dallas, Texas USA; Departments of Biophysics and Biochemistry, University of Texas Southwestern Medical Center, Dallas, Texas USA; Howard Hughes Medical Institute, University of Texas Southwestern Medical Center, Dallas, Texas USA

**Keywords:** *Stachybotrys*, Comparative genomics, Secondary metabolism, Trichothecene biosynthesis, Toxins, Satratoxins, Atranones, Whole-genome sequencing

## Abstract

**Background:**

The fungal genus *Stachybotrys* produces several diverse toxins that affect human health. Its strains comprise two mutually-exclusive toxin chemotypes, one producing satratoxins, which are a subclass of trichothecenes, and the other producing the less-toxic atranones. To determine the genetic basis for chemotype-specific differences in toxin production, the genomes of four *Stachybotrys* strains were sequenced and assembled *de novo*. Two of these strains produce atranones and two produce satratoxins.

**Results:**

Comparative analysis of these four 35-Mbp genomes revealed several chemotype-specific gene clusters that are predicted to make secondary metabolites. The largest, which was named the core atranone cluster, encodes 14 proteins that may suffice to produce all observed atranone compounds via reactions that include an unusual Baeyer-Villiger oxidation. Satratoxins are suggested to be made by products of multiple gene clusters that encode 21 proteins in all, including polyketide synthases, acetyltransferases, and other enzymes expected to modify the trichothecene skeleton. One such satratoxin chemotype-specific cluster is adjacent to the core trichothecene cluster, which has diverged from those of other trichothecene producers to contain a unique polyketide synthase.

**Conclusions:**

The results suggest that chemotype-specific gene clusters are likely the genetic basis for the mutually-exclusive toxin chemotypes of *Stachybotrys*. A unified biochemical model for *Stachybotrys* toxin production is presented. Overall, the four genomes described here will be useful for ongoing studies of this mold’s diverse toxicity mechanisms.

**Electronic supplementary material:**

The online version of this article (doi:10.1186/1471-2164-15-590) contains supplementary material, which is available to authorized users.

## Background

*Stachybotrys* is a genus of filamentous fungi found in soil worldwide [1]. It can also inhabit damp buildings. It is mainly a saprophyte that feeds by degrading cellulose and other dead plant matter. However, it is related to cellulolytic plant pathogens including *Fusarium* and *Myrothecium*, and there is a report of soybean invasion [2]. *Stachybotrys* has never been reported to infect animals. However, it does produce a variety of toxins that have killed livestock and sickened humans after contact with contaminated feed (reviewed in [3]).

Some recent studies have suggested links between *Stachybotrys*-infested damp buildings and poor health. For example, *Stachybotrys* infestation was correlated with a cluster of infant hemosiderosis in Cleveland in the 1990s [4], and several case studies have found relationships between mold-infested buildings and poor health (reviewed in [3]). However, as yet there is no consensus on specific symptoms associated with long-term low-level exposure to *Stachybotrys*, and any environmental study of its impact is difficult. One reason for this is that *Stachybotrys* rarely infests buildings in isolation, but rather is found with other toxigenic and allergenic mold species [3]. Another is that *Stachybotrys* can produce potentially beneficial compounds such as the antiviral stachyflins [5] and a cyclosporin immunosuppressant [6]. In addition, *Stachybotrys* products have been shown *in vitro* to include both proteins, *e.g.,* proinflammatory proteases [7] and antigenic proteins [8], and also secondary metabolites [9].

**Figure 1:**
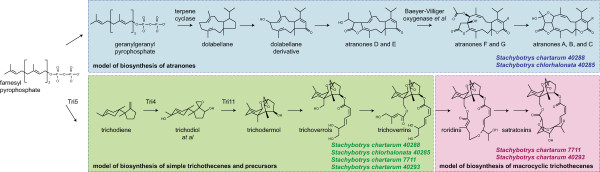
**The two toxin chemotypes of** ***Stachybotrys.*** Both atranones and satratoxins are terpenoid secondary metabolites thought to derive from the primary metabolite farnesyl pyrophosphate (FPP). Box colors indicate each class of molecule and its specific secondary metabolite precursors: blue for atranones, green for simple trichothecenes, and pink for macrocyclic trichothecenes, which include satratoxins. *Atranones* are diterpenoids thought to originate from cyclization of geranylgeranyl pyrophosphate to form dolabellane, which has an eleven-membered ring [11]. Shown are the structures of all atranones solved by Hinkley *et al.*[11], as well as types of enzymes capable of catalyzing the two postulated reactions in the pathway. *Trichothecenes* are sesquiterpenoids that are products of FPP cyclization. The pathway of trichodermol biosynthesis from FPP is known experimentally [12, 13], but there are no experimental data regarding biosynthesis pathways of satratoxins or other trichodermol derivatives. Shown is a conceptual pathway adapted from [14] and references therein. It integrates results from several trichothecene producers. Enzymes shown have been functionally characterized from *Fusarium* (Tri5) or *Trichoderma* (Tri4 and Tri11). Trichodiol is shown to represent several intermediates that undergo both enzymatic hydroxylation and spontaneous rearrangement to form trichodermol, which is the first molecule shown that contains the trichothecene skeleton, *i.e.,* the tricyclic ring 12,13-epoxytrichothec-9-ene (EPT). In *Fusarium,* trichodermol is not observed. Instead, the pathway after trichodiol diverges into a series of products substituted at C-3 of EPT. There are two known trichoverrols (A and B) and two known trichoverrins (A and B), but the respective pairs differ only in the stereochemistry of the C-4 side chain. The satratoxin F/G skeleton is shown as representative of satratoxins, and roridin E as representative of roridins. Omitted for brevity are the verrucarins (double arrow between roridins and satratoxins).

The two most well-known classes of secondary metabolite toxins are the trichothecenes and the atranones (Figure 1). Both are terpenoids, but they are not otherwise related in structure. The more toxic class, trichothecenes, is further divided into two subclasses, simple and macrocyclic trichothecenes, with the latter subclass including the highly-toxic compounds called satratoxins (intranasal LD_50_ ~ 1 mg/kg in rodents [1]). Of the ~200 strains of *Stachybotrys* that have been tested, all can make simple trichothecenes [10]. However, only a third of these strains can make macrocyclic trichothecenes (*e.g., satratoxins*). Of the other two-thirds, most can make the less-toxic atranones. In fact, these strains are the only known atranone-producing organisms. A strain of *Stachybotrys* that makes both satratoxins and atranones has never been observed, suggesting that these chemotypes are mutually exclusive. The hypothesis of the current study was that these two divergent phenotypes are due to the presence of strain-specific secondary metabolite gene clusters in *Stachybotrys.*

To determine the genetic basis for the two chemotypes of *Stachybotrys* and to compare *Stachybotrys* to other trichothecene toxin producers including *Fusarium* and *Trichoderma,* the genomes of four cultured *Stachybotrys* strains were sequenced and assembled *de novo*. Two of these strains make atranones, and the other two make satratoxins. Some global properties of these genomes are reported, most notably their richness of polyketide synthase (PKS) genes. The core trichothecene cluster (CTC) of *Stachybotrys* is presented and shown to diverge significantly from the CTCs of other trichothecene producers, with a genomic context that appears to be chemotype-specific. Finally, comparative methods are used to support the hypothesis that the toxin chemotype in *Stachybotrys* may arise from the presence of strain-specific secondary metabolite biosynthesis gene clusters, including three satratoxin chemotype-specific clusters and a novel 35-kbp locus that has been named the core atranone cluster (CAC).

## Results and discussion

### Sequencing and assembly of *Stachybotrys*genomes

**Figure 2:**
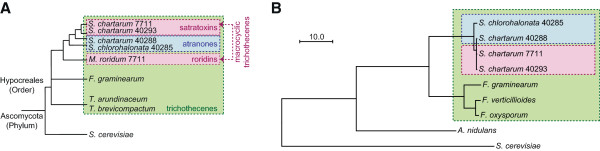
**Conceptual and ortholog-based maximum likelihood phylogeny of** ***Stachybotrys*** **and other fungi. A**. The conceptual phylogeny shows the toxin chemotypes of the four sequenced *Stachybotrys* strains in relation to other trichothecene-producing fungi of order *Hypocreales. S. cerevisiae* is only distantly related to *Hypocreales* and is shown for context. Topology adapted from [18]. **B**. Phylogeny was constructed from alignment of 2,177 proper protein orthologs identified by OrthoMCL. Scale bar shows number of substitutions per site. All branches have 100% support.

The phylogeny of the four *Stachybotrys* strains that were sequenced is shown in Figure 2A. The strains include two species, *S. chlorohalonata* (IBT strain 40285) and *S. chartarum* (IBT strains 40288, 40293, and 7711), which are distinguishable both by morphology and molecular markers. Strains 40285 and 40288 make atranones, while strains 40293 and 7711 make satratoxins (Figure one; [15]). The genomes of these four strains were obtained by massive parallel sequencing on an Illumina Hiseq 2000. For each strain, a separate 300-bp nominal genomic fragment library was constructed. These libraries were multiplexed in order to combine them all on a single sequencer lane. Sequencing yielded ~70 million 101-bp reads per strain after demultiplexing and error correction. Each genome was then independently assembled with SOAPdenovo [16], followed by protein annotation of each assembly with MAKER [17] using a cross-strain iterative strategy. Ideally these annotations would be supported by RNA data, but the RNA extractable from each of the four strains was too degraded to use for RNA-seq libraries, preventing this additional validation.

Table 1 summarizes the genome and proteome assemblies, and for comparison also includes a finished assembly of the trichothecene producer *Fusarium graminearum* obtained by Sanger sequencing [19]. These five genome and proteome assemblies are similar in size, although those of the *S. chlorohalonata* strain 40285 are slightly smaller than the three *S. chartarum* strains. Except for the N_50_ length, the features of all four *Stachybotrys* assemblies, *e.g.,* their short introns and sparse repeat content, are comparable to the finished *F. graminearum* assembly. This is consistent with the fact that *Fusarium* is known to be closely related to *Stachybotrys* [18]. Each strain was independently assembled with ABySS [20] to validate the SOAPdenovo results. While scaffold N_50_ length obtained from ABySS was reduced by 20 to 80-kbp versus scaffold N_50_ length from SOAPdenovo, total genome sizes were nearly identical. Also, the seven gene clusters described below for the SOAPdenovo build were appropriately present in the ABySS assemblies. Specifically, in both the ABySS and SOAPdenovo assemblies, the core trichothecene cluster had identical architecture in all four strains, and the other six novel clusters described were consistently either atranone- or satratoxin-specific.

**Table 1:** **Features of** ***Stachybotrys*** **genome and proteome assemblies**

	***S. chlorohalonata 40285***	***S. chartarum 40288***	***S. chartarum 40293***	***S. chartarum 7711***	***F. graminearum PH-1***
**NCBI Acc. #**	APWP00000000	AQPQ00000000	ASEQ00000000	APIU00000000	AACM00000000
**Paired reads [×10** ^**6**^ **]**	66.4	58.6	68.8	71.4	NA
**Assembled sequences**	1246	957	826	897	36
**Assembly size [Mbp]**	34.2	36.5	36.1	36.2	36.2
**Fold coverage**	196	162	192	199	10
**N**_**50**_ **length [kbp]**	116	130	214	177	5350
**Assembly gaps [Mbp]**	0.25	0.08	0.16	0.13	0.22
**Repeat content [%]**	1.62	0.93	0.93	1.01	0.66
**Gene content [%]**	51.75	53.42	53.19	53.31	57.18
**Coding genes [predicted]**	10866	11719	11532	11543	13332
**Median gene length [bp]/protein length [aa]**	1357/403	1377/411	1380/412	1379/413	1259/375
**Mean exons per gene**	2.8	2.8	2.8	2.8	2.8
**Median: exon length [bp]/intron length [bp]**	293/59	296/59	297/59	296/59	255/55
**Predicted products with identified CDD domain [%]**	65.87	65.84	66.29	65.94	61.43

*Stachybotrys* assemblies include all contigs and scaffolds of at least 1-kbp. N_50_ is the sequence that includes the middle nucleotide of the assembly when the sequences are ordered by length.

Comparative proteome content of *Stachybotrys* Two methods were used to estimate the completeness of the *Stachybotrys* proteome assemblies and to compare them to those of other sequenced fungi. First, CEGMA [21] was used to search the *Stachybotrys* genome assemblies for 458 proteins known to be highly conserved in eukaryotes. By this criterion, each assembly is 98% complete, with identical completeness found for *F. graminearum* and the other two sequenced *Fusarium* genomes, *F. oxysporum* and *F. verticillioides,* neither of which make trichothecenes. All proteins found by CEGMA were independently found by MAKER in the full *Stachybotrys* proteomes, suggesting that the *Stachybotrys* genome assemblies are relatively complete.

Second, groups of homologs in the proteome assemblies were identified with OrthoMCL [22]. For diversity, nine proteomes were used: the four *Stachybotrys* assemblies, the three *Fusarium* proteomes named above [19], and two more divergent model fungi: *Aspergillus nidulans* [23] and *Saccharomyces cerevisiae* [24]. OrthoMCL clustered these proteomes into 16,311 groups, each containing at least two proteins. Of these groups, 2,177 contained exactly one orthologous sequence from each of the nine proteomes. Using this subset of proper orthologs, a robust phylogeny was constructed (Figure 2B) and proteome divergence was quantified by calculating pairwise sequence identities (Table 2). The phylogeny matches both accepted taxonomy and a previous molecular phylogeny [18], validating both the predicted *Stachybotrys* proteomes and the OrthoMCL-based method used to identify homologs. As expected given prior analysis of *Stachybotrys* genetic markers [15], the proteome identities indicate that the *S. chlorohalonata* strain 40285 is the most divergent of the four *Stachybotrys* strains. However, this divergence is relative, because there is 98% proteome identity between 40285 and any *S. chartarum* strain, 74% identity between *Stachybotrys* and *Fusarium,* and >99% identity within the three strains of *S. chartarum*.

**Table 2:** **Ortholog-based pairwise proteome identities of** ***Stachybotrys*** **and other fungi**

	***7711***	***40293***	***40288***	***40285***	***Fve***	***Fox***	***Fgr***	***Ani***	***Sce***
***7711***	100	99.830	99.746	97.701	73.668	73.646	72.995	54.834	39.231
***40293***		100	99.742	97.707	73.663	73.644	72.998	54.836	39.231
***40288***			100	97.673	73.663	73.649	73.000	54.836	39.237
***40285***				100	73.667	73.638	73.011	54.832	39.240
***Fve***					100	97.174	89.068	55.506	39.796
***Fox***						100	89.380	55.452	39.742
***Fgr***							100	54.934	39.373
***Ani***								100	39.740
***Sce***									100

The proteome abbreviations in the table represent four organisms sequenced in this study: *7711* – Stachybotrys *chartarum 7711*, *40293* – *Stachybotrys chartarum 40293*, *40288* – *Stachybotrys chartarum 40288*, and *40285* – *Stachybotrys chlorohalonata*. Proteomes of other fungi included in the analysis are: *Fve, Fusarium verticillioides; Fox, Fusarium oxysporum; Fgr, Fusarium graminearum; Ani, Aspergillus nidulans; Sce, Saccharomyces cerevisiae.*

**Figure 3:**
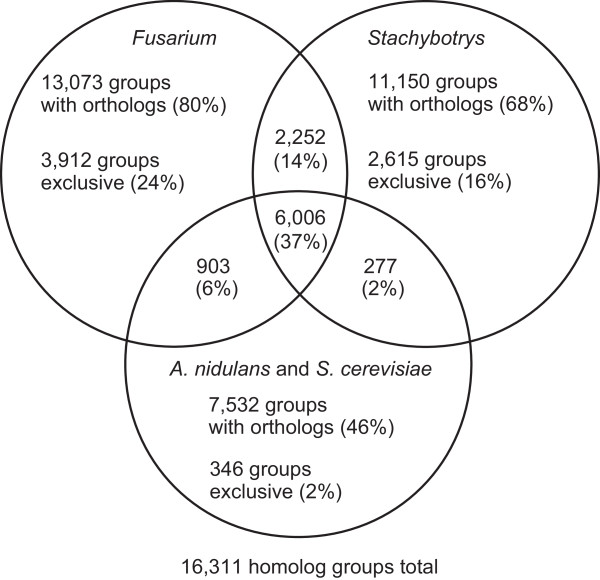
**Distribution of orthologs of** ***Fusarium*** **and** ***Stachybotrys.*** This Venn diagram shows the number of protein homolog groups, out of a 16,311 total, in each combination of three sets: (1) groups with a homolog in any *Stachybotrys* genome; (2) groups with a homolog in any *Fusarium* genome; and (3) groups with a homolog in *A. nidulans* or *S. cerevisiae* which for simplicity are pooled as a single outgroup.

Figure 3 summarizes the distribution of homolog groups in the four genera. Of the 16,311 homolog groups, most included orthologs from *Stachybotrys* (68% of all groups) and *Fusarium* (80%). Many groups were exclusive to *Stachybotrys* (16% of all groups) or *Fusarium* (24%). Most of the proteins in the 2,615 groups exclusive to *Stachybotrys* lack known domains (only 37% contain at least one domain from the Conserved Domain Database (CDD [25]), versus ~65% of all *Stachybotrys* proteins). A similarly low fraction (39%) of *Fusarium-*exclusive proteins include a CDD domain, so this result is likely not an artifact of the annotation method.

To infer functional trends in proteins exclusive to *Stachybotrys,* domain enrichment analysis was performed (Additional file 1). This revealed that of the *Stachybotrys*-exclusive protein domains, those enriched relative to the domains of non-exclusive *Stachybotrys* proteins likely have specialized functions such as mating enforcement (the CDD HET domain), degradation of plant materials (glycosyl hydrolases Glyco_hydro_61 and Glyco_hydro_6; several peptidase domains including M28; the pectate lyase domain Amb_all; and the cellulose-binding domain fCBD), and synthesis of novel secondary metabolites or other products (methyltransferases, acetyltransferases, and cytochrome P450 monooxygenases). The whole domain compositions of the *Stachybotrys* and *Fusarium* proteomes were also compared independently of homology considerations (Additional file 1). Domain enrichment analysis revealed that only nine domains out of the 5,752 tested are differentially present between the two genera. Four CDD domains are enriched in the *Stachybotrys* proteome relative to *Fusarium.* Two of them, fCBD and Glyco_hydro_61, are also enriched in the *Stachybotrys*-exclusive proteins described above. The other two domains, PKS and PKS_AT, are respectively the ketosynthase and acyltransferase domains that are found constitutively in type I iterative polyketide synthases (PKSs). In fungi, PKSs are large proteins of variable domain architecture that are responsible for producing a diverse array of polyketide secondary metabolites [26]. Each strain of *S. chartarum* conservatively encodes 35–37 PKSs (Additional file 2), which is more than any other known fungus and over twice as many as *Fusarium.* This suggests that a multitude of secondary metabolites from *Stachybotrys* remain uncharacterized. PKSs also appear to play roles in *Stachybotrys’s* biosynthesis of trichothecenes and atranones.

### The core trichothecene gene cluster of *Stachybotrys*diverges from those of other trichothecene producers

Many fungal secondary metabolites are made by products of genes that are found adjacent to one another in a single contiguous locus [27]. These genetic loci are known as secondary metabolite biosynthesis (SMB) clusters. SMB clusters throughout the *Stachybotrys* assemblies were identified with the antiSMASH cluster prediction software [28]. Each assembly contains 50–70 SMB clusters (Additional file 3), with predicted end products including polyketides, nonribosomal peptides, and other classes.

**Figure 4:**
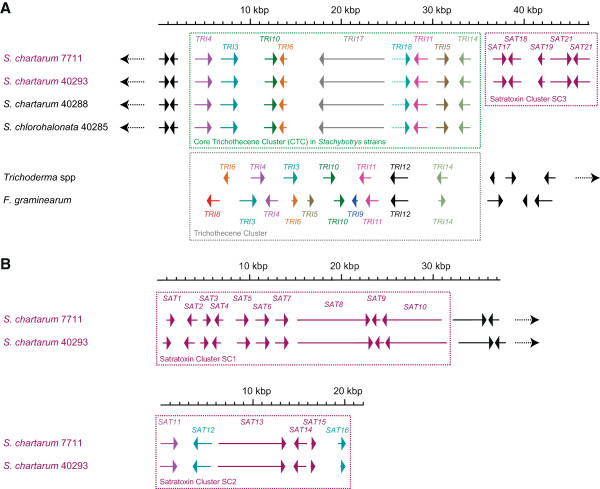
**The core trichothecene clusters of** ***Stachybotrys, Trichoderma,*** **and** ***F. graminearum,*** **and satratoxin chemotype-specific clusters SC1, SC2, and SC3 of** ***Stachybotrys.*** **A**. The core trichothecene cluster (CTC). For all genomes an arrow indicates a gene and its transcriptional sense. The core trichothecene clusters of *Stachybotrys* are shown in the green box, and the adjacent satratoxin cluster SC3 is shown in the pink box. The other genes that are shown outside the boxes lack similarity to known trichothecene synthesis genes, so they are assumed to be in flanking regions outside these two clusters. A black, dotted arrow indicates that a scaffold extends to include other genes beyond the region shown, whereas lack of such an arrow indicates a scaffold border. The color indicates orthology with respect to *Trichoderma* and *F. graminearum* trichothecene clusters (shown in the gray, dotted box). *TRI18*, which is a paralog of *TRI3*, is colored as TRI3, but the arrow is dotted. Note that *Trichoderma TRI5* is known to exist outside of the CTC [12]. The ruler at the top indicates length in kbp. *Trichoderma* and *F. graminearum* CTCs were redrawn from prior work [12, 29]. **B**. The satratoxin-specific clusters are shown in the pink boxes. The other genes shown are chemotype-independent. Other figure conventions follow those described for the CTC.

In the simple trichothecene producers *Fusarium graminearum* and *F. sporotrichioides*, the core trichothecene gene cluster (CTC) is a well-studied example of an SMB cluster (Figure 4A). The *Fusarium* CTC encodes 11—12 genes, most of which are required to catalyze specific steps in trichothecene production [29]. CTC sequences are also available for *Trichoderma arundinaceum* and *T. brevicompactum* [12]. However, each of these organisms’ CTC encodes only seven genes. This divergence of the *Fusarium* and *Trichoderma* CTCs reflects the biochemical divergence of trichothecene pathways between the genera. Most prominently, *Fusarium* makes only products modified at backbone position C-3, such as deoxynivalenol and T-2 toxin. In contrast, *Trichoderma* does not modify C-3, but exclusively makes trichothecenes modified at backbone position C-4, including trichodermol (Figure one; [30]).

Each of the *Stachybotrys* assemblies includes a complete and identical ~30-kbp locusinferred to be the *Stachybotrys* CTC (Figure 4A)*.* This CTC was manually defined to comprise nine genes, including putative orthologs of seven *Fusarium* and *Trichoderma* genes: the terpene cyclase *TRI5,* the acetyltransferase *TRI3*, the hydroxylases *TRI4* and *TRI11,* the transcription factors *TRI6* and *TRI10,* and a gene of unknown function *TRI14.* The remaining two genes in the *Stachybotrys* CTC are novel, so they were named by convention: the putative PKS *TRI17,* and adjacent to it the *TRI3* paralog *TRI18.*

The patterns of proteins coded in the *Fusarium, Trichoderma,* and *Stachybotrys* CTCs (Figure 4A) are consistent with both the divergence of the *Stachybotrys* CTC from that of *Fusarium* and the similar gene content of the *Stachybotrys* and *Trichoderma* CTCs, which share six genes. However, the divergence in *Stachybotrys* gene order from the *Trichoderma* CTC was unexpected, since the initial trichothecenes made by *Stachybotrys* and *Trichoderma* are identical. For example, unlike in *Trichoderma,* where the *TRI5* gene is located outside of the CTC [12] *, Stachybotrys TRI5* is located within the CTC. There is a single syntenic block between the two taxa containing *TRI4, TRI3,* and *TRI10,* and the relative positions of *TRI11* and *TRI14* are also conserved*.* However, conservation of these relationships does not extend to *Fusarium* (Figure 4A).

Two additional results support the novel CTC architecture of *Stachybotrys*. First is the fact that two independent genome assemblers yielded the same sequence. Second is the fact that the recently-sequenced CTC of the macrocyclic trichothecene producer *Myrothecium roridum* (Figure 2) has a similar architecture, including mostly-conserved gene order and the presence of putative *TRI17* and *TRI18* orthologs (Robert H. Proctor, personal communication; unpublished data). The diversity of the CTC (Figure 4A) is consistent with the hypothesis that it is a hotspot for insertion and deletion of enzyme-coding genes, in turn allowing for substantial structural diversity of trichothecenes.

Most *Stachybotrys* paralogs of *Fusarium* and *Trichoderma* trichothecene synthesis genes are found within the *Stachybotrys* CTC. However, also identified are two *Stachybotrys* loci outside of the CTC that contain paralogs of *Stachybotrys* CTC genes. First, there is the satratoxin chemotype-specific cluster SC2 (Figure 4B), which contains paralogs of *TRI3* and *TRI4*. Second, the assembly of strain 40293 includes a small scaffold (not shown) that contains only two genes. They have been named *TRI19* and *TRI20* and are paralogs of *TRI5* and *TRI6,* respectively. *Stachybotrys* orthologs of other known *Fusarium* trichothecene biosynthesis genes have not been identified in these assemblies. In particular, the trichothecene exporter *TRI12*, which is present in the CTCs of both *Fusarium* and *Trichoderma* [12], is absent in *Stachybotrys*.

The results of the antiSMASH run were compared to the manual definition of the CTC (Additional file 4). In each strain, AntiSMASH detected a single gene cluster that included (1) all nine of the CTC genes that had been defined manually; (2) the five genes specific to satratoxin-producing strains (SC3, discussed below); (3) three additional genes ~2-kbp upstream; and (4) two genes 5-kbp downstream of the CTC genes defined manually. The three upstream genes have orthologs in *F. verticillioides* and *F. oxysporum,* but they lack known orthologs in *F. graminearum, M. roridium,* or any other trichothecene producer. Therefore, they were omitted from the initial definition of the *Stachybotrys* CTC. However, they contain oxidoreductase, aldo-keto reductase, and glutathione S-transferase domains, all of which are known or thought to have roles in SMB [27, 31]. Thus, it is possible that these three proteins do participate in synthesis of trichothecenes specific to *Stachybotrys.* The two genes 5-kbp downstream are unique to *S. chorohalonata* 40285. Neither of the predicted proteins includes a CDD domain, and in both cases BLASTP yields only hits to hypothetical proteins from *F. oxysporum* and other *Hypocreales* (E > 1e-60 and 1e-16, respectively). Thus, they were not included in the definition of the *Stachybotrys* CTC.

**Figure 5:**
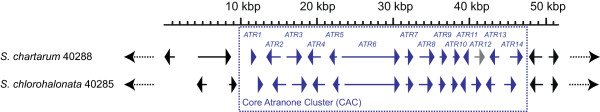
**The core atranone clusters of the** ***Stachybotrys*** **atranone-producing strains.** The core atranone clusters are shown in the blue box. The other genes shown are chemotype-independent. *ATR12* of strain 40288 is gray to indicate that it is a possible pseudogene, because despite its translation having ~90% identity to 40285 Atr12, in the present assembly its exon 1 contains an internal stop codon.

The products of the core atranone cluster likely suffice to make all known atranone species. The hypothesis of this study was that the two mutually-exclusive chemotypes of *Stachybotrys* were due to the presence of strain-specific SMB clusters. To test this hypothesis computationally, the four *Stachybotrys* genome assemblies were searched for loci that were present in both satratoxin strains but in neither atranone strain, or *vice versa*. The custom search strategy combined two methods, both based on sequence alignment. At the genomic level, four-way whole-genome alignment was employed, using Mugsy [32]. At the level of the proteome, the sets of homologs compiled with OrthoMCL were considered. Whole-genome alignment was needed to show genomic context, but in practice Mugsy aligned some locus boundaries incorrectly, so its results were manually adjusted as described in the Methods. Overall, the search yielded a total of two atranone-specific and four satratoxin chemotype-specific gene clusters. The larger of the two atranone-specific gene clusters was named the core atranone cluster (CAC, or AC1; Figure 5, Additional file 5). This is a ~35-kbp PKS-based cluster, and it has a nearly-identical architecture of 13–14 genes (*ATR1-ATR14*) in both atranone strains. The CAC is complete in the sense that the genes immediately flanking it on both sides are not atranone-specific.

It is predicted that the products of the CAC catalyze most or all steps of atranone synthesis, starting from geranylgeranyl pyrophosphate (GGPP; Figure 1). This prediction is based on two observations. First, the CAC is one of only two clusters exclusive to two relatively divergent strains of *Stachybotrys.* Second, the predicted CAC products satisfy some key constraints of the chemical model for atranone biosynthesis (Figure 1) proposed by Hinkley *et al.* [11]. The Hinkley model includes two characteristic reactions: the initial cyclization of GGPP to dolabellane and a Baeyer-Villiger oxidation near the end of the atranone synthesis, which converts atranones D and E to atranones F and G. In the CAC, the initial cyclization could be performed by the predicted terpene cyclase product of *ATR13*. This prediction is based on the presence of the terpene cyclase motif DDXXE [27] in ATR13 and its high similarity to fungal terpene cyclases (the best BLAST hit has E < 1e-40). The Baeyer-Villiger oxidation could be performed by the predicted product of *ATR8.* This protein contains the FXGXXXHXXXWD motif, which is specific to Bayer-Villiger monooxidases (BVMOs) [33]. The high similarity of ATR8 to the BVMO phenylacetone monooxygenases from fungi (the best BLAST hit has E < 1e-65) strengthens the argument. Although terpene cyclases are relatively common in the four *Stachybotrys* proteomes, the BVMO motif is very rare. There is only one other set of homologs that contain the BVMO motif. However, this second set of putative BVMOs has representatives in all four strains, and OrthoMCL groups it separately from the atranone-specific pair found in the CAC in a chemotype-independent cluster that contains only glycosyl hydrolases, suggesting that its function is not chemotype-specific. Taken together, all these data support the hypothesis that the CAC’s products function to synthesize atranones.

Of the CAC’s other predicted gene products, the largest is the reducing PKS Atr6 (Figure 5 and Additional file 5)*.* A BLAST search suggests that this protein is related to both fungal and bacterial PKSs, with the best hit to an uncharacterized PKS from *Aspergillus fumigatus.* Some other predicted CAC products include four oxygenases, three short-chain reductases, an esterase, and a methyltransferase. These may all be involved in the various steps of atranone biosynthesis, although their specific roles must await experimental determination because the types of reactions that they catalyze appear frequently in the Hinkley model (Figure 1).

AntiSMASH’s definition of the CAC included all 14 of the genes identified by the alignment-based search method described above (Additional file 5), plus two additional genes, one on either flank (coordinates in Additional file 3). The first additional gene is 2-kbp upstream of the CAC definition and has an ortholog in all four *Stachybotrys* strains. It contains an ANK domain, and has BLASTP hits to hypothetical fungal proteins (E > 1e-22). The second additional gene is 2-kbp downstream of the CAC definition and has an ortholog in strains 40285, 40288, and 40293. It contains a DOMON_DOH domain, found in monooxygenase proteins, and has a strong BLASTP hit to a putative monooxygenase in the grapevine pathogen fungus *Togninia minima* (E = 0, 75% identity, 98% coverage). Although it is possible that these two proteins have roles in atranone biosynthesis, they were not included in the initial CAC definition because they are not specific to the atranone strains.

If the CAC’s gene products truly function to synthesize atranones as suggested by this analysis, then how atranone biosynthesis is regulated remains an open question. No transcription factors or other putative regulatory genes have been identified within the CAC or nearby. The closest sequence coding a chemotype-independent *GAL4*-family gene is 21-kbp upstream. Other examples of fungal SMB clusters that lack internal regulatory genes are the alkaloid clusters of the epichloae [34] and the penicillin cluster of *Aspergillus nidulans* and other species [35]. However, a scan of the 14 putative CAC promoter regions revealed that 20 of the 28 promoter regions contain the palindromic sequence AGACATGTCT, which suggests that a yet-unidentified transcription factor is involved in the CAC regulation. Additionally, some CAC products may be widely expressed and post-transcriptionally regulated. It was reported that most atranone-producing *Stachybotrys* strains easily produce simple dolabellane derivatives in culture, but do not always produce atranones [10], which is consistent with the hypothesis that some enzymes in this pathway are not regulated at the level of transcription.

The second atranone-specific gene cluster is named AC2 (Additional file 5). It is smaller than the CAC, spanning 12-kbp and containing six genes, and was not identified by antiSMASH. Unlike the CAC, AC2 lacks any genes at one flank in the assemblies, so it may be incomplete. Also, three of its genes are homologous to those of a second distinct locus conserved in all *S. chartarum* strains (on scaffold645 of 40288, scaffold1203 of 40293, and scaffold1305 of 7711). The largest gene in AC2 putatively encodes the phosphate transporter domain PHO4, and another encodes an HLH transcription factor. Two other genes yielded relatively weak BLAST hits (E ≈ 1e-4 in both cases) to cyclins and arrestins, suggesting overall that AC2 could be related to environmental phosphate sensing. Because phosphate-substituted compounds are used in the synthesis of terpenes, specifically-regulated phosphate transport may be necessary for appropriate production of FPP or other atranone precursors. Unfortunately, it is not yet possible to obtain a confirmation via genetics due to the lack of systems for genetic manipulation and recombination in *Stachybotrys.*

### Gene clusters specific to satratoxin-producing strains of *Stachybotrys*

A general biosynthesis model for the satratoxins has been proposed, based on the known structures of similar molecules (Figure one, adapted from [14]). In this model, satratoxins and all other macrocyclic trichothecenes derive from trichodermol, first by sequential esterification of two side chains to C-4 and C-15 hydroxyl groups on the trichothecene skeleton, and second by condensation of the two side chains to form the macrocycle. Based on their structures, the side chains may be polyketide products, although they would need to be modified by external hydroxylases to yield the primary hydroxyl groups observed. PKS-independent reductases and methyltransferases may also be involved.

The whole-genome comparative method revealed four satratoxin chemotype-specific gene clusters, three of which encode the types of enzymes required for satratoxin synthesis (Table 3, with genome coordinates in Additional file 6). They are named satratoxin clusters (SCs) 1–4, in order of size. The two largest, SC1 and SC2 (Figure 4B), are classical PKS-based SMB clusters. SC3 (Figure 4A) is smaller and is not a complete SMB cluster on its own, but it is found adjacent to the CTC. As shown in Figure 4A and 4B, all three SCs are at the borders of their respective scaffolds, which raises the possibility that they are located close to the CTC and can thus be easily co-regulated.

**Table 3:** **Summary of functions putatively encoded by genes in satratoxin clusters SC1, SC2, and SC3**

Symbol	Exons	Putative functions	Closest homolog	E-value/Identity [%]	Conserved domain database
SAT1-7711 SAT1-40293	1	Membrane protein, FAD binding protein, contains domain found in fungal squalene epoxidases and monoxygenases	Putative FAD binding domain-containing protein from *Eutypa lata.*	5e-58/49	cl19134 cl17314
SAT2-7711 SAT2-40293	2	NADPH-dependent short-chain dehydrogenase/reductase	Hypothetical protein from *Setosphaeria turcica*.	5e-124/53	cd05327
SAT3-7711 SAT3-40293	2	Classical short-chain reductase	Putative short chain dehydrogenase reductase protein from *Eutypa lata*	5e-124/67	cd05233
SAT4-7711 SAT4-40293	3	Putative integral membrane protein	Putative integral membrane protein from *Eutypa lata*.	7e-89/54	No conserved domains detected
SAT5-7711 SAT5-40293	1	Putative acetyltransferase	Putative trichothecene 3-o- protein *Eutypa lata.*	93-165/54	cl19241
SAT6-7711 SAT6-40293	1	A secretory lipase domain	Related to lipase 1 from *Fusarium fujikuroi*	1e-166/53	cl14925
SAT7-7711 SAT7-40293	1	Squalene epoxidase	Putative salicylate hydroxylase from *Aspergillus ruber*	5e-132/46	cl17314
SAT8-7711 SAT8-40293	3	Putative polyketide synthase with a conventional non-reducing architecture	Putative polyketide synthase from *Aspergillus ruber*	0.0/48	cd00833
SAT9-7711 SAT9-40293	2	Putative Cys6 transcriptional factor	Hypothetical protein from *Sordaria macrospora*	2e-25/33	No conserved domains detected
SAT10-7711 SAT10-40293	6	Putative protein containing four ankyrin repeats	Multiple ankyrin repeats single kh domain protein from *Metarhizium anisopliae*	5e-78/25	cd00204
	7				
SAT11-7711 SAT11-40293	5	Putative cytochrome P450 monooxygenase and a Tri4 paralog	Cytochrome P450 from *Myrothecium roridum*	0.0/78	cl12078
SAT12-7711 SAT12-40293	5	Putative 15-O-acetyltransferase Tri3	Hypothetical protein from *Endocarpon pusillum*	1e-91/36	cl06457
SAT13-7711 SAT13-40293	2	Putative reducing polyketide synthase	Putative polyketide synthase protein from *Eutypa lata*	0.0/57	cd00833
SAT14-7711 SAT14-40293	1	Sat14 and Sat16 are complete and truncated paralogs of the acetyltransferase Tri3	Predicted protein from *Nectria haematococca*	0.0/67	pfam13523
SAT15-7711 SAT15-40293	2	The zinc finger protein Sat15	LolU from *Epichloe festucae*	8e-25/38	No conserved domains detected
SAT16-7711 SAT16-40293	4	Sat14 and Sat16 are complete and truncated paralogs of the acetyltransferase Tri3	Trichothecene 15-O-acetyltransferase from *Fusarium graminearum*	9e-36/45	cl06457
	3				
SAT17-7711 SAT17-40293	5	TauD hydroxylase	Hypothetical protein from *Tuber melanosporum*	5e-67/39	pfam02668
SAT18-7711 SAT18-40293	4	Methyltransferase	Hypothetical protein from *Cladophialophora psammophila*	4e-35/28	cl16913
SAT19-7711 SAT19-40293	2	N-acetyltransferase	Hypothetical protein from *Nectria haematococca*	4e-25/30	cd04301
SAT20-7711 SAT20-40293	3	Cys6-type zinc finger	Hypothetical protein from *Macrophomina phaseolina*	3e-43/30	No conserved domains detected
	4				
SAT21-7711 SAT21-40293	6	MFS (Major Facilitator Superfamily)-type transporter	Hypothetical protein *Gaeumannomyces graminis*	2e-94/37	pfam07690

For brevity the closest homologs, their E-values and levels of identity are identified only for *Stachybotrys chartarum* 7711.

SC1 (Figure 4B and Table 3) is a 30-kbp cluster that contains ten genes, *SAT1-SAT10.* The largest genes are *SAT8,* which encodes a putative PKS with a conventional non-reducing architecture [26], and *SAT10*, which encodes a putative protein containing four ankyrin repeats (RPS-BLAST prediction) and thus may be involved in protein scaffolding. The putative short-chain reductase Sat3 may assist the PKS in some capacity. Sat6 contains a secretory lipase domain and is similar to the *Fusarium* trichothecene C-15 esterase Tri8 (BLASTP E-value 3e-93, 40% identity, 85% coverage), although it shows even greater similarity to other uncharacterized proteins from *Fusarium* (BLASTP E-value 1e-151, 52% identity, 87% coverage) and *Aspergillus* (BLASTP E-value 1e-101, 41% identity, 86% coverage). The adjacent gene *SAT5* encodes a putative acetyltransferase, and so the two together may effect endogenous protection from toxicity in the same manner as Tri8 and Tri101 of *Fusarium* [36].

AntiSMASH identified all ten genes in SC1 (Additional files 3 and 4). It also defined the orthologous clusters as containing 4–5 additional genes: two downstream genes present in all three of the *S. chartarum* strains, two genes (one upstream and one downstream) present only in strain 7711, and one satratoxin chemotype-specific gene located 1-kbp downstream of *SAT10* (Additional file 4). This flanking gene putatively encodes a 1,061-aa protein that contains a Peptidases_S8_S53 domain and has BLASTP hits only to hypothetical fungal proteins (E > 1e-90). This gene was not included in the initial definition of SC1 because there is no known function for peptidases in secondary metabolism.

**Figure 6:**
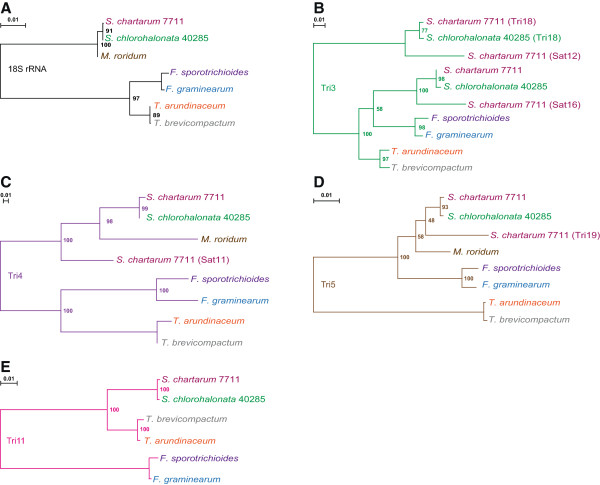
**Maximum likelihood phylogenies of selected Tri homologs. A**. Reference phylogeny made from partial 18S rRNA sequences. **B**. Tri3, including all four *Stachybotrys* paralogs from CTC and SC2. **C**. Tri4, including *Stachybotrys* paralog from SC2. **D**. Tri5, including the paralog Tri19 from strain 40293. **E**. Tri11. Each phylogeny is rooted at the midpoint. Branches in each tree are colored by gene using the same scheme as in Figure 4. Each organism is labelled with a separate color. Branches are labelled with support values of 100 total bootstrap replicates. Scale bars show the number of substitutions per site.

SC2 is 20-kbp and contains six genes, *SAT11-SAT16,* the largest of which encodes the putative reducing PKS Sat13 (Figure 4B and Table 3). The alignment-based method is in agreement with antiSMASH on this cluster definition (Additional file 6). SC2 is unique among the gene clusters described here because three of its genes are paralogs of genes from the CTC cluster (relationships shown in Figures 4 and 6). Sat11 is a cytochrome P450 monooxygenase and a Tri4 paralog, while Sat14 and Sat16 are complete and truncated paralogs of the acetyltransferase Tri3, respectively. Finally, the cluster may be regulated by the zinc finger protein Sat15, which is most similar (BLASTP E-value 7e-25, 38% identity, 94% coverage) to the LolU protein reported from an SMB cluster of the grass-endophytic fungus *Neotyphodium* [34, 37]. Only six putative LolU homologs were identified in *Stachybotrys* 7711, and one also flanks the CTC of *M. roridum* (Robert H. Proctor, personal communication). Taken together with the novel architecture of the *Stachybotrys* CTC, these data suggest that SC2 may have originated as a duplication of the CTC and has subsequently undergone rearrangements and divergence in function.

In contrast to SC1 and SC2, SC3 (Figure 4A and Table 3) is a small 10-kbp cluster that contains five genes, *SAT17-SAT21*. AntiSMASH agrees with the alignment-based method on this definition of SC3 (Additional file 4). Although none of the genes in SC3 encode a PKS, the cluster itself is found adjacent to the CTC in satratoxin strains (Figure 4A), suggesting that the two loci may be co-regulated. One of the SC3 genes, *SAT21,* encodes a putative MFS-type transporter which may have a role in exporting secondary metabolites (Table 3). In *Fusarium*, the transporter protein Tri12 exports simple trichothecenes [38], but is unrelated in sequence to Sat21. The four other proteins putatively encoded in SC3 include the TauD hydroxylase Sat17, the methyltransferase Sat18, the acetyltransferase Sat19, and the Cys6-type zinc finger Sat20 (Table 3). Sat20 may be involved in regulation of SC3, as there is evidence for Cys6-type zinc finger regulation of other fungal SMB clusters [39, 40].

The smallest satratoxin chemotype-specific locus, SC4, is a region that is located in the middle of a chemotype-independent gene cluster. It does not appear to encode any of the types of enzymes described above, nor any other enzymes known to be involved in terpene synthesis. It also was not identified by antiSMASH. As it is difficult to predict the function of SC4, it has been included in Additional file 6 mainly for formal completeness. In comparison to the atranone case, no unusual chemistry has been proposed for the biosynthesis of satratoxins that would more specifically inform as to the relevance of any of these four chemotype-specific loci. Indeed, given the recent divergence of the satratoxin strains relative to the atranone strains (Table 2), it is possible that SC4 or other clusters identified here are unrelated to satratoxin biosynthesis and are specific to these two satratoxin strains only by chance. This is a fundamental limitation of both the experimental design of this study and the comparative method in general. As with the CAC, it will eventually be necessary to verify the function of these clusters experimentally. At the same time, a straightforward comparative experiment to test and refine the satratoxin model presented here would be to search for these satratoxin chemotype-specific clusters in the genome of *Myrothecium roridum,* a more divergent macrocyclic trichothecene producer (Figure 2A) that does not yet appear to be fully sequenced.

### Phylogenies for four trichothecene biosynthesis protein families in *Stachybotrys,*and functional implications

Four well-studied CTC proteins are Tri5, Tri4, Tri11, and Tri3. Tri5, which cyclizes FPP to trichodiene, and Tri4, which hydroxylates trichodiene and its derivatives in multiple positions, are the earliest known enzymes in the trichothecene pathway (Figure one; [30]). Both Tri4 and Tri11 are known to catalyze different reactions in *Fusarium* versus *Trichoderma* [12], resulting in two genus-specific series of trichothecenes (C-3 vs non-C-3 substituted). To infer the functions of these genes in *Stachybotrys* and more generally to explore the evolution of the CTC and SC2, maximum likelihood-based phylogenies were constructed of these four proteins and their paralogs (Figure 6). These phylogenies included homologs from *Stachybotrys*, *Myrothecium* (only Tri5 and Tri4 are available), *Trichoderma*, and *Fusarium*. Partial 18S rRNA sequences are available for all four genera [18], and these were used to construct a reference phylogeny (Figure 6A). Excluding the *Stachybotrys* SC2 products and other paralogs, the topology of the 18S tree matches that of Tri4 (Figure 6C) and Tri3 (Figure 6B). However, the 18S tree differs from that of Tri5 (Figure 6D), in which *Trichoderma* Tri5 is divergent, and Tri11 (Figure 6E), in which *Fusarium* Tri11 is divergent. The Tri5 topology may result from the fact that in *Trichoderma,* TRI5 is located outside of the CTC [12]. The Tri11 topology is consistent with *Stachybotrys* Tri11 conserving the function of *Trichoderma* Tri11, which is to hydroxylate the trichothecene skeleton at C-4 to yield trichodermol [12]. Although no functional prediction for *Stachybotrys* Tri4 can be made based only on this tree, it is assumed that similar to the Tri4 from *Trichoderma*, its product lacks the ability to hydroxylate C-3, since C-3 substituted trichothecenes have not been observed in *Stachybotrys* [30].

Three of the four tree topologies in Figure 6 (Tri5, Tri4, and Tri3) mostly match the topologies of 18S (Figure 6A), which may support a single origin for the CTC in the common ancestor of all four genera. However, in the *Stachybotrys* paralogs, the 18S topology is conserved for the Tri5 paralog Tri19 (Figure 6D), but not for the Tri4 paralog Sat11 (Figure 6C), which diverges before *Myrothecium* Tri4, nor for the Tri3 paralogs Tri18 and Sat12 (Figure 6B), which form the outgroup to all Tri3 and Sat16. These results are consistent with gene duplication or independent horizontal transfer events occurring prior to *Stachybotrys* speciation. Furthermore, the clustering of Tri3 with Sat16 and Tri18 with Sat12 in Figure 6B is consistent with the hypothesis that the satratoxin chemotype-specific cluster SC2 originated as a duplication of the CTC.

Why are the chemotype-specific gene clusters mutually exclusive? The above analyses suggest that the presence of certain gene clusters may suffice to produce the strain-specific products observed in *Stachybotrys.* However, the mechanism or selection pressures by which these clusters have come to be mutually exclusive remain unclear. Chemotype mutual exclusivity in *Stachybotrys* is not well-explained either by chance or by geographic isolation, because the chemotypes of ~200 *Stachybotrys* strains are known [1], and there is no relationship between chemotype and geographic location. For instance, three of the strains reported here were isolated from the San Francisco Bay Area with two of these, the atranone strain 40285 and the satratoxin strain 40293, acquired from the same apartment unit [41]. This study also contradicts the hypothesis that both chemotypes have all the machinery needed to produce both atranones and satratoxins, but there is a strain-specific metabolic shunt at work minimizing production of one type of toxin or the other. It is possible that by unknown mechanisms, the presence of the atranone cluster and a strain’s susceptibility to satratoxin toxicity are linked. One way to test this would be to transfect the CAC into a satratoxin strain and observe colony growth. However, currently this experiment is not feasible due to the lack of an appropriate model system. It is also possible that there is some novel regulatory mechanism at work that prevents the inclusion of both sets of clusters in a single strain.

## Conclusions

**Figure 7:**
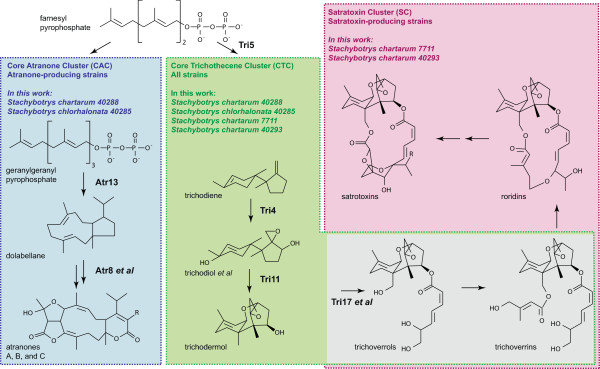
**Unified genetic model for atranone and satratoxin biosynthesis.** Molecules are color-coded per Figure 1. The gray box indicates trichothecenes whose catalysis is uncertain; they may be synthesized by enzyme products of the core trichothecene cluster, by products of satratoxin chemotype-specific clusters, or by a mix of both types.

The findings of this study are summarized with a unified genetic model for atranone and satratoxin biosynthesis (Figure 7) that also incorporates much previous work by biochemists [11, 12, 14, 30]. Some aspects of this model are speculative, such as the location of the boundary between trichothecenes produced by atranone strains and those produced by satratoxin strains. Although atranone strains are known to make trichodermol, it is unknown whether they can make early macrocyclic trichothecene intermediates such as trichoverrols and trichoverrins. Due to the presence of the chemotype-independent PKS gene *TRI17* within the CTC, it is speculated that atranone strains can produce trichoverrols, though perhaps not trichoverrins. An assay of this chemotype in atranone-producing strains will be critical to more precisely determine the functions of the putative satratoxin chemotype-specific enzymes identified in this study.

## Methods

### *Stachybotrys*culture, DNA extraction, and library construction

*Stachybotrys* strains were kindly provided by Kristian F. Nielsen (Center for Microbial Biotechnology, DTU, Denmark). Fungus was grown on potato dextrose agarose to establish monoclonal populations by single-spore selection. These monoclonal populations were used for all subsequent procedures. Strain identities were verified by PCR-based sequencing of *TRI5* [15]. For sequencing libraries, hyphae were grown in 3-ml tubes of potato dextrose broth at 25°C in the dark for 1–2 weeks until confluent. Genomic DNA for sequencing libraries was obtained by a method based on cetyltrimethylammonium bromide (CTAB) disruption and phenol-chloroform extraction [41]. Fresh hyphae were drained of media and pulverized in liquid N_2_. The sample was added to a tube containing hot 2x CTAB buffer and n = 3 5-mm glass beads, and then bead-beaten on a vortexer for 1 mn. DNA was extracted with 25:24:1 phenol:chloroform:isoamyl alcohol, treated with Riboshredder (Epicentre) for 30 minutes at 37°C, and precipitated with isopropanol.

Multiplexed Illumina DNA fragment libraries were constructed as follows. For each strain, 500–1000 ng genomic DNA was sheared by sonication (Bioruptor, Diagenode) to ~500 bp. Fragments were end-repaired (NEBNext End Repair Module, NEB), dA-tailed (NEBNext dA-Tailing Module, NEB), and ligated (NEBNext Quick Ligation Module, NEB) to custom Y-adapters that included strain-specific 4- or 5-bp barcodes. Each reaction product was purified with Agencourt AMPure XP beads (Beckman). Ligated product was size-selected to 350 bp (nominal) by electrophoresis on 2% agarose, excision, and gel extraction (MinElute Gel Extraction kit, Qiagen) overnight at room temperature. Following size selection, each library was amplified by PCR (Phusion High Fidelity PCR Master Mix with GC Buffer, NEB) using the standard Illumina primers, with 3 ng template, 0.5 μM primers, 12 PCR cycles per reaction, and other reagents and reaction parameters per NEB’s instructions. All PCRs in this study were performed on a PCR Express thermal cycler (Thermo Hybaid). PCR product was size-selected as above to remove unreacted primer and adapter dimers. The four libraries were then pooled to 2.5 nM each and submitted to the UT Southwestern Genomics Core for sequencing on a single lane of an Illumina HiSeq 2000.

### Genome assembly and resequencing of specific loci

Base-calling of reads from intensity data was accomplished with AYB 2.11 [42]. This yielded 394 million paired reads, 72% of which passed purity filtering. Pure reads were demultiplexed and sequencing artifacts, including reads containing adapter and primer sequence, were removed using custom scripts. The remaining reads were end-trimmed to quality 20 or higher. Reads were spectrally corrected with Quake 0.3.0 [43] and then assembled *de novo* into contigs and scaffolds with SOAPdenovo 1.05 [16] and AbySS 1.3.4 [20]. For each strain and assembler, n = 27 (SOAPdenovo) or n = 10 (AbySS) separate assemblies were produced, in each case iterating K from 31 to 81. A single assembly with a subjectively good combination of total size and N_50_ length was then selected as the representative final assembly; these two parameters were generally robust over a wide range of K values. The final SOAPdenovo assemblies had the following K values: strain 40285, K = 43; strain 40288, K = 53; strain 40293, K = 45; and strain 7711, K = 51.

Two loci discussed in Results are each split over two different sequences in the assemblies: the CTC of strain 40293 and the CAC of strain 40288. Sanger sequencing of PCR amplicons verified that each of these regions is in fact a single contiguous locus, although in each case the two flanking regions are separated by an estimated 50—100 bp repeat that has not proven possible to sequence by either the parallel or the Sanger method. The PCR primers used were as follows. For CTC, primer pair 1: forward TTGGTCGTCTCTTGAGATTCACTGGC, reverse CCAAAGTGGAAGGTTCATGGTTGAGC; primer pair 2: forward TTCCCTTGCTTCCGTACCTTATTCCC, reverse TTATTCCCATCCTTTGTCCGGAGTGG. For CAC, primer pair 1: forward AAGTCTCATCTTGCCTCGGAATCAGG, reverse AGTTCAACCTTCTCTCAGGAACAGGG; primer pair 2: forward CCTGATCTTGGACATTGCTATTCCGC, reverse TTTGCATGAGCTAAACACACCGGG. The CTC was amplified in a 50 μl reaction including 5 μl Accuprime Pfx reaction mix, 0.4 μl Accuprime Pfx DNA polymerase, 0.3 μM each primer, and 3 ng genomic DNA from strain 40293. The CAC was amplified in a 100 μl reaction including 10 μl Accuprime Pfx reaction mix, 0.8 μl Accuprime Pfx DNA polymerase, 0.3 μM each primer, and 3 ng genomic DNA from strain 40288. PCR parameters included 30 (CTC) or 35 (CAC) cycles of denaturation at 95°C for 15 s, annealing at 55°C (CTC) or 58°C (CAC) for 30 s, and extension at 68°C for 60 s. Before sequencing, both products were gel purified (Minelute Gel Extraction Kit, Qiagen), reamplified with the same PCR parameters as were used for the first reaction, and repurified (Wizard SV kit, Promega).

### Proteome assembly

For proteome assembly (*i.e.,* protein annotation), MAKER 2.26 [17] was used. MAKER incorporated both homology-based (BLAST 2.2.26 and Exonerate 2.2.0) and *de novo* methods (GeneMark 2.3e and Augustus 2.6.1) and output only transcript models that were supported by both types of evidence. For each strain, MAKER was run twice. The second pass was run in reannotation mode, and included as homology targets all four proteomes output by the first pass. On both passes, the other homology targets included the Swissprot database (20 Aug 2012 build) and the three *Fusarium* proteomes [19]. Full input parameters for MAKER are listed in Additional file 7.

For the comparison shown in Table 1, features of the *F. graminearum* genome were obtained from the *Fusarium graminearum* Genome Database [44].

### Genetic nomenclature of *Stachybotrys*and data availability

In naming *Stachybotrys* genes and proteins, the conventions in use for *E. coli* and *Fusarium* were followed*.* All gene and protein names are three letters followed by a number. Gene names are all-uppercase and italicized, *e.g.*, “*TRI5”*. Corresponding protein names are capitalized and in standard face, *e.g.,* “Tri5”.

### Protein and rRNA phylogenies

To construct Figures 2B and 6, proteins were downloaded from NCBI using the accessions listed in the cited references. Following protein alignment with the L-INS-i method of mafft 6.903b [34], any position containing a gap was discarded. Protein phylogenies were inferred with PhyML 20120412 [35], using 100 bootstrap replicates and otherwise default parameters.

### Proteome comparisons and SMB cluster inventory

To obtain groups of homologous proteins, OrthoMCL 2.0 [22] was run on nine proteomes using default parameters, including a BLAST E-value cutoff of 1e-5. Proteome identities were calculated between each pair of genomes by finding the pairwise sequence identities of all 2,177 proper orthologs, *i.e.,* OrthoMCL groups that contained exactly one orthologous sequence from each of the proteomes.

Protein domains were identified by searching the nine proteomes with RPS-BLAST 2.2.26 against the NCBI Conserved Domain Database (CDD; 2 Aug 2012 version), and then filtering results using the NCBI Specific Hits algorithm [25]. Domain enrichment analysis is described in the caption to Additional file 1. All domain identifiers mentioned in the Results are the unique “domain short names” assigned by CDD. To identify SMB clusters in the assemblies and to verify the custom alignment-based method of finding chemotype-specific gene clusters, antiSMASH 2.0 [28] and SMURF [45] were run via the authors’ Web servers, using the default parameters. A putative *Stachybotrys* PKS was defined as any predicted protein that includes all three of the CDD domains PKS, PKS_AT, and either PKS_PP or PP-binding.

Identification of chemotype-specific gene clustersA *chemotype-specific gene cluster* was defined as a locus containing at least three genes, all of which are both chemotype-specific and contiguous. This definition implies that a cluster’s boundaries (or flanks) correspond to the end of the region specific to both strains in the chemotype. Chemotype-specific gene clusters were identified by collating OrthoMCL homolog sets with chemotype-specific loci found by whole-genome alignment with Mugsy 1r2.2 [32]. Initially a custom Python script (available at <http://prodata.swmed.edu/jrs/maf-stachy2/>) was run on the whole-genome alignment to identify all *candidate clusters.* A candidate cluster was defined as a subalignment of at least 100-bp that was either (1) present in both satratoxin strains but in neither atranone strain (“satratoxin-specific”), or (2) present in both atranone strains but in neither satratoxin strain (“atranone-specific”). After identification of candidate clusters, OrthoMCL results were manually inspected to exclude those regions that were not chemotype-specific gene clusters as defined above and to manually adjust the boundaries of clusters that did meet this definition. For example, Mugsy sometimes failed to align local regions that had repetitive sequences, thus incorrectly splitting some chemotype-specific alignments. These regions were joined manually, and they were then verified to be chemotype-specific by a local BLASTN search. Conversely, at the boundaries of chemotype-specific loci, Mugsy sometimes included regions that were not in fact chemotype-specific, as judged by the OrthoMCL results. Thus, the boundaries of these loci were manually adjusted to include only chemotype-specific genes.

### Availability of supporting data

These four genome and proteome assemblies have been deposited at NCBI as Bioproject PRJNA186748, <http://www.ncbi.nlm.nih.gov/bioproject/186748>. The four Genbank accessions are listed in Table 1.

## Electronic supplementary material

Additional file 1: **Domains enriched in the** ***Stachybotrys*** **proteome.** Lists of *Stachybotrys* protein domains that are significantly enriched (corrected p-value <0.001) in comparison to control sets, by Fisher’s exact test. Sheet 1 lists domains that are enriched in proteins exclusive to *Stachybotrys,* relative to all *Stachybotrys* domains. Sheet 2 lists domains that are overrepresented in the entire set of *Stachybotrys* proteins, relative to the entire set of *Fusarium* proteins. A positive log_2_ odds ratio indicates that the domain is overrepresented in the test group relative to the control; conversely, a negative odds ratio indicates underrepresentation. The P-values shown were corrected for multiple testing with the Bonferroni method. (XLSX 13 KB)

Additional file 2: **Putative polyketide synthases of** ***Stachybotrys.*** Sheet 1 provides the total counts of putative PKSs found in sequenced fungal genomes. The counts include hybrid NRPS/PKSs. Counts were computed for *Stachybotrys, Fusarium* spp., and *A. nidulans;* others are reprinted from Table eight of [46]. Sheets 2–5 list the putative PKSs and NRPS/PKSs of *Stachybotrys* strains 40285, 40288, 40293, and 7711, including transcript ID, length, and predicted domain architecture, respectively. Sheet 6 shows the number of PKSs, NRPS, and terpene cyclases in each strain. (XLSX 15 KB)

Additional file 3: **Secondary metabolite biosynthesis gene clusters in the four** ***Stachybotrys*** **assemblies.** This file lists all secondary metabolite biosynthesis gene clusters predicted by antiSMASH. For each cluster, columns include parent sequence name, cluster number, type of predicted metabolite product, and start and end coordinates. (XLSX 20 KB)

Additional file 4: **Summary of genes in CTC and SC3.** For each gene ortholog in each strain, the columns show the following data: gene symbol if assigned; transcript ID; contig or scaffold in which the gene is found; one-based coordinates; length of the gene in nt; strand designation on the contig or scaffold; number of exons; length of putative product in aa; whether the gene was identified as part of a chemotype-specific locus by the custom method based on genome alignment and ortholog analysis; whether the gene was identified as part of an SMB cluster by antiSMASH; whether the gene was identified as part of an SMB cluster by SMURF; and the Stachybotrys strains with orthologs, as identified by OrthoMCL: either specific strain names, 40285 and 40288 (“atranone”), 40293 and 7711 (“satra”), or all four strains (“4xStachy”). (XLSX 18 KB)

Additional file 5: **Summary of genes in the two atranone-specific clusters of strains 40288 and 40285.** For description of file, see caption of Additional file 4. (XLSX 15 KB)

Additional file 6: **Summary of genes in satratoxin chemotype-specific clusters SC1 and SC2 of strains 40293 and 7711.** For description of file, see caption of Additional file 4. (XLSX 16 KB)

Additional file 7: **Parameters for genome annotation.** This file concatenates the two parameter files used by MAKER during the second and final pass of our annotation. These specific files were used for strain 7711, but parameters were the same for the other three assemblies. (TXT 6 KB)
